# Simultaneous Estimation of Azimuth and Elevation Angles Using a Decision Tree-Based Method

**DOI:** 10.3390/s23167114

**Published:** 2023-08-11

**Authors:** Anabel Reyes Carballeira, Felipe A. P. de Figueiredo, Jose Marcos C. Brito

**Affiliations:** National Institute of Telecommunications INATEL, Av. João de Camargo, 510-Centro, Santa Rita do Sapucaí 37540-000, MG, Brazilbrito@inatel.br (J.M.C.B.)

**Keywords:** direction of arrival, machine learning, correlation matrix, decision tree, music

## Abstract

This study addresses the problem of accurately predicting azimuth and elevation angles of signals impinging on an antenna array employing Machine Learning (ML). Using the information obtained at a receiving system when a transmitter’s signal hits it, a Decision Tree (DT) model is trained to estimate azimuth and elevation angles simultaneously. Simulation results demonstrate the robustness of the proposed DT-based method, showcasing its ability to predict the Direction of Arrival (DOA) in diverse conditions beyond the ones present in the training dataset, i.e., the results display the model’s generalization capability. Additionally, the comparative analysis reveals that DT-based DOA estimation outperforms the state-of-the-art MUltiple SIgnal Classification (MUSIC) algorithm. Our results demonstrate an average reduction of over 90% in the prediction error and 50% in the prediction time achieved by our proposal when compared to the MUSIC algorithm. These results establish DTs as competitive alternatives for DOA estimation in signal reception systems.

## 1. Introduction

In signal processing, Direction of Arrival (DOA) denotes the direction of signals impinging on a sensor or antenna array [1]. DOA estimation methods have been investigated for decades due to their actual application in radar, sonar, seismology, astronomy, and military surveillance. Furthermore, DOA has become more significant due to the development of mobile networks, technologies, and devices. For example, Beamforming (BF) is a technology that is becoming more important every day in mobile communication networks [2]. BF is a technique that focuses a wireless signal toward a specific receiving device rather than having the signal spread in all directions from an omnidirectional antenna, as it usually would. In this way, the received signal level is increased. Thus, it is essential to know the device’s direction in order to to precisely align the antenna’s beam with it.

On the other hand, the wide number of drone devices in agriculture, industry, transport, communication, surveillance, etc., along with the development of the Internet of Things (IoT), means that the use of such devices is increasing considerably [3,4]. However, drones can seriously threaten civilian environments or sensitive areas such as airports and military bases. For example, in January 2015, a drone crashed at the White House [5], compromising the security of the government building. On 29 March 2016, a Lufthansa jet came within 200 feet and collided with a drone near Los Angeles International Airport [6]. On 12 October 2017, a Beech King Air A100 of Skyjet Aviation collided with an Unmanned Aerial Vehicle (UAV) as the former approached Jean Lesage Airport near Quebec City, Canada. The aircraft landed safely despite being hit on the wing [7].

Due to the security risk associated with drones, as aforementioned, it is necessary to implement drone DOA systems to locate and confiscate or disable them. For the DOA system to work, a drone’s signal is required in order to discover its directions. However, drone owners do not want to be discoverable, and they will not transmit such a signal that makes them vulnerable to detection or localization through a DOA system. Fortunately, most drones transmit signals to their controllers to send images, video, and telemetry reports. Another important use case for a DOA system is the localization and recovery of drones that lost contact with their controllers. These drones generally keep sending data to their controllers. Therefore, these signals can be used by DOA systems to find the drone’s direction.

In 5G and 6G wireless networks [8], the estimation of the DOA plays a crucial role across various applications. One of its key benefits lies in facilitating the deployment of smart antenna systems, allowing adaptive steering of antenna arrays to optimize both signal reception and transmission [9]. Moreover, DOA information finds utility in localization and positioning applications, as it enables accurate triangulation of user device locations by estimating the DOA of signals from multiple base stations or access points [10]. Another notable application of DOA estimation pertains to enhancing network security, as the analysis of signal DOAs permits the detection and mitigation of spoofing attacks or unauthorized signal sources [11]. Furthermore, DOA estimation offers valuable insights for network planning and optimization processes, aiding in efficient resource allocation and improved network performance [12].

Hence, due to the importance of the DOA estimation, a new method, based on ML, for finding the azimuth and elevation angles of a signal impinging on a receiving antenna array system is proposed and analyzed in this paper.

The remainder of this paper is organized as follows. In Section 2, we discuss several DOA estimation methods and related works. Section 3 describes the system model used to collect the information that feeds the ML model. Section 4 describes our proposal based on an ML model for the DOA estimation that predicts both the azimuth and elevation angles. Simulation results are presented and discussed in Section 5. We draw the limitations of our proposal in Section 6. Finally, in Section 7, we present our conclusion.

## 2. Related Works

Different studies have been carried out to establish the DOA of an incoming signal [13,14,15,16,17,18,19,20,21,22,23,24,25,26,27,28,29,30,31,32,33,34,35,36,37,38,39,40,41,42]. DOA estimation techniques can be broadly classified into conventional beamforming techniques, Maximum Likelihood Estimator (MLE), and Subspace-Based Techniques (SBT). However, there are recent works on Received Signal Strength (RSS)-based DOA estimation [43]. Moreover, in recent years, ML has been applied to this problem. This section will briefly summarize these techniques and their principal works, which introduce ML to obtain better DOA results.

### 2.1. Maximum Likelihood Estimation

The MLE finds its estimates by maximizing the probability density function of the observed received signals concerning the model’s parameters. MLE solutions can be classified into two approaches: stochastic MLE and deterministic MLE. In the stochastic MLE, the signals are assumed to be Gaussian distributed [13,14,15]. Deterministic MLE methods consider the signals as being arbitrary and deterministic [15,16]. The sensor noise is modeled as Gaussian in both methods, which is a reasonable assumption due to the central limit theorem. As a result, the stochastic MLE achieves the Cramer-Rao Bound (CRB). On the other hand, this does not hold true for deterministic MLE methods [17].

The problem with MLE techniques is the high computational cost involved since they have to solve a nonlinear multidimensional optimization problem for which global convergence is not guaranteed [18].

### 2.2. Subspace-Based Techniques

The best-known subspace-based methods are MUSIC [19] and Estimation of Signal Parameters Via Rotational Invariance Techniques (ESPRIT) [20], on which many works have been based. MUSIC-based methods employ the orthogonality of the signal subspace (steering vectors) and the noise subspace to search the spatial spectrum to achieve high resolution. ESPRIT-based methods exploit an underlying rotational invariance among signal subspaces induced by an array of sensors with a translational invariance structure. When the uncertainty of the system or background noise leads to model errors, e.g., the wrong number of sources, subspace-based methods need to solve high-dimensional non-linear parameter estimation problems. Many improved algorithms based on MUSIC and ESPRIT have been developed to estimate the number of sources [21,22]. However, these approaches must sacrifice the array aperture and deteriorate the resolution to deal with the singular matrix of the spatial covariance issue.

### 2.3. Sparse Signal Reconstruction

The Sparse Signal Reconstruction (SSR) technique has been used in DOA estimation [23,24,25,26,27]. It exploits the property that the spatial spectrum of the point source signals is sparse when the number of signals is limited. The key is to use appropriate non-quadratic regularizing functions (such as $\ell_{p}$-norms), which lead to sparsity constraints and super-resolution. In addition, the primary concern in the SSR technique lies in the computational complexity.

### 2.4. Machine Learning

ML is presented as a promising technology to be used for DOA estimation. ML-based methods are data-driven and more robust than other methods due to their adaptability to the array geometry and sensor imperfections. They also do not depend on the array geometry shape [44]. In addition, ML offers low-cost implementation and simplicity.

The authors in [28,29,30,31,32,33,34,35,36] used a Neural Network (NN) for DOA estimation. The authors in [28] proposed an azimuth estimation method using a Complex Valued Neural Network (CVNN) for ultra-wideband systems, where the received signal feeds the CVNN. The authors validate their proposal via simulation and experiments. The results are compared to MUSIC and Real-Valued Neural Networks (RVNNs). In [29], a Multilayer Perceptron Neural Network (MPNN) is presented that can learn from a large amount of simulated noisy and reverberant microphone array inputs for robust DOA estimation. Specifically, the MPNN learns the nonlinear mapping between Generalized Cross-Correlation Vectors (GCC) features and DOA. In [30], the authors used a Deep Neural Network (DNN) for DOA estimation. They evaluated the estimation performance under a scenario where two equal-power and uncorrelated signals are incidents on a Uniform Linear Array (ULA). The authors in [31] focused on scenarios where the number of active sources may exceed the number of simultaneously sampled antenna elements. For this purpose, they proposed new schemes based on NN and estimators that combine NNs with gradient steps on the likelihood function. The authors in [32] propose a cascaded neural network consisting of the SNR classification and the elevation angle estimation for two closely spaced sources. The authors used the correlation matrix as the input of a DNN for DOA estimation. In [33], the authors integrate Multiple Input Multiple Output (MIMO) systems with DNN for channel estimation and DOA determination of a source. In this work, good results are obtained in the simulations. However, they only focus on the azimuth angle and require a complex system (e.g., in their simulation, the radio base of the MIMO system is equipped with 128 antennas). In [34], the authors propose a new DOA method based on an ML model to estimate the azimuth angle of a signal. A dataset named “Dround Data New” was obtained with a four-antenna-based system that contains well-known received powers. The authors trained and validated the dataset with a DNN model. In [35], the authors presented a deep ensemble learning to find a source’s azimuth and elevation angles to different training conditions. The authors designed a Convolutional Neural Network (CNN) that performs the regression task to learn a mapping between the spatial covariance matrix of the received signals from the antenna elements and the DOA. In addition, to improve the prediction performance, they proposed an ensemble learning method. Simulation results show that their proposals have slightly lower Mean Squared Error (MSE) performance than conventional methods such as MUSIC and $\ell_{1}$-norm SVD (Singular Value Decomposition). However, the simulation results also show that the authors’ proposals in this article respond very fast and have higher speeds than the MUSIC and $\ell_{1}$-norm SVD algorithms. In [36], the authors proposed Circularly Fully Convolutional Networks (CFCN) to find the DOA of multiple sources in low-frequency scenarios. The CFCN is trained using the dataset labeled with space-frequency pseudo-spectra and accompanied by on-grid DOA predictions. Then, the regression model is developed to estimate the precise DOAs according to corresponding proposals and features.

In [37,38,39,40,41,42], the authors proposed a Support Vector Regression (SVR)-based DOA estimation method. In [37], a smart antenna system is considered to estimate the DOAs of multiple sources in noiseless and noisy environments.In [38], the authors address the problem of estimating the DOAs of coherent electromagnetic waves incident on a ULA, building upon previous work and presenting experimental results. In [39], a multi-resolution approach for the real-time DOA estimation of multiple signals impinging on a planar array is presented. The method is based on a Support Vector Classifier (SVC), and it exploits a multi-scaling procedure to enhance the angular resolution of the detection process in the regions of incidence of the incoming waves. The authors in [40] proposed the combination of the advantages of Forward–Backward Linear Prediction (FBLP) and SVR in estimating DOAs of coherent incoming signals with low snapshots. In [41], the authors proposed a scheme to address the wideband DOA estimation problem. An approach for the real-time DOA estimation of multiple signals impinging on a planar array is presented in [42]. This last work estimates the azimuth and elevation angles of one or more incident sources in a ULA. However, the angular resolution is shallow. The main disadvantage of Support Vector Machine (SVM) is the algorithm’s time complexity [45]. The algorithmic complexity of SVM models affects the model training time on large datasets, the development of optimal models for multiclass, and the performance on unbalanced datasets.

Table 1 summarizes the articles mentioned above that employ ML techniques to solve the DOA problem. The table shows the main aspects considered to carry out our work. In addition, the table collects the angles considered by the related works: azimuth, elevation, or both. It also shows if the work is oriented to find the DOA from a single source or several, if the proposals were validated via simulation or experiments, and if the results were compared with an existing DOA model.

ML has been mainly used to improve the estimation of azimuth or elevation angles and computing speed. In addition, various studies have been carried out to find the origin of multiple sources. However, the study of ML-based DOA for estimating azimuth and elevation angles has yet to be deeply studied. The reason behind this is the huge size of the training data, which are only supported by some models such as NNs [46]. However, estimating the elevation and the azimuth angles is crucial and has many applications in various engineering fields. For instance, with complete DOA information, it is possible to improve the transmission coverage in wireless communications by avoiding interference and enhancing the system capacity [42]. More specifically, the knowledge of the azimuth and elevation angles would enable more effective utilization of the beamforming (BF) technology in the next generation of mobile networks. Therefore, this study addresses the problem of accurately predicting the azimuth and elevation angles of a signal impinging on an antenna array.

The main goal of this work is to propose a simple, in terms of computational complexity, yet accurate ML model for DOA estimation that predicts both the azimuth and elevation angles. To achieve this goal, the following specific objectives are considered:

- Design a receiving antenna system to estimate the DOA.
- Propose an ML-based DOA estimation solution capable of adapting to different conditions, such as the SNR of the signal.
- Discuss training data preparation and design for a specific scenario.
- Optimize the ML-based DOA estimation solution.
- Compare the results obtained with the ML-based DOA estimation solution with a state-of-the-art DOA estimation method present in the literature.

## 3. System Model

**Figure 1:**
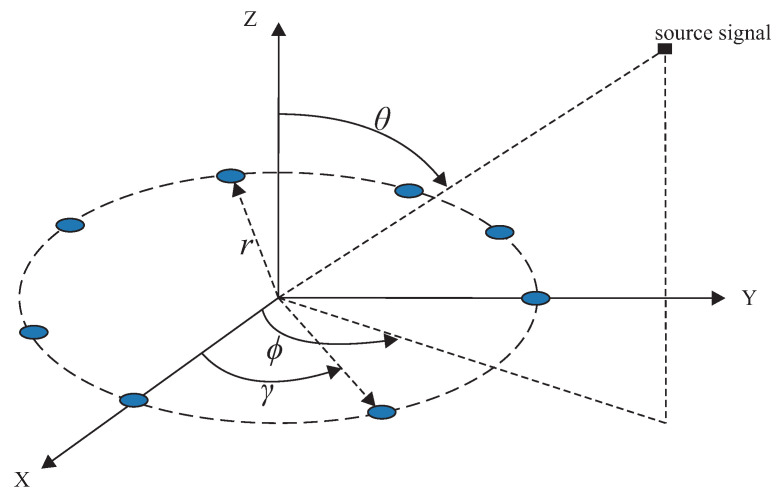
Receiver system.

We consider a receiving system that consists of a single-channel receiver and *M* half-wavelength strip dipole antennas operating at a frequency of 70 MHz (70 MHz is the maximum frequency supported by the computer used for the simulations in terms of processing power) [47,48]. Therefore, only one antenna is connected to the receiver at a time. The *M* antennas are uniformly placed along a circle of radius, r=λ/2, forming a Uniform Circular Array (UCA) as shown in Figure 1, where λ is the incident signal’s wavelength.

If a signal impinges on the center of the circle formed by the *M* antennas with azimuth, ϕ, and elevation, θ, angles as shown in Figure 1, the received signal at the *m*-th antenna element can be mathematically given as

(1) $$y_{m}\left(k\right)=a_{m}\left(\phi ,\theta ,\gamma_{m}\right)s\left(k\right)+n_{m}\left(k\right),$$

where m∈{1,…,M} is the antenna index, k∈{1,…,N} is the sample index, $a_{m}$(ϕ, θ, $\gamma_{m}$) represents the attenuation factor (amplitude attenuation and phase shift) of the *m*-th antenna, *s(k)* is the *k*-th signal transmitted by the source as it arrives at the antenna array and $n_{m}$*(k)* is the *k*-th noise sample at the *m*-th antenna, which has its value drawn from a circularly symmetric (central) complex normal distribution with variance equal to $\sigma^{2}$. The attenuation factor, $a_{m}$(ϕ, θ, $\gamma_{m}$), is expressed as

(2) $$a_{m}\left(\phi ,\theta ,\gamma_{m}\right)=e^{\frac{-j2\pi r\cos\left(\gamma_{m}-\phi \right)\cos\left(\theta \right)}{\lambda }},$$

where *r* is the radius of the receiving system, and $\gamma_{m}$ is the angular position of the antenna array elements, which can be calculated as

(3) $$\gamma_{m}=\frac{2\pi (m-1)}{M}.$$

Therefore, the received signal vector obtained at the output of the antenna array can be written as

(4) $$y\left(k\right)={\left[y_{1}\left(k\right),y_{2}\left(k\right),\ldots ,y_{M}\left(k\right)\right]}^{T},$$

which can be re-expressed as

(5) y(k)=As(k)+n(k),

where ${\left[\cdot \right]}^{T}$ denotes the transpose and **A**, **s**(k), and **n**(k) are given by

(6) $$\begin{array}{c} A=\left[\begin{array}{ccc} e^{\frac{-j2\pi r\cos\left(\gamma_{1}-\phi \right)\cos\left(\theta \right)}{\lambda }} & \ldots & 0\\ \vdots & \ddots & \vdots \\ 0 & \ldots & e^{\frac{-j2\pi r\cos\left(\gamma_{M}-\phi \right)\cos\left(\theta \right)}{\lambda }} \end{array}\right], \end{array}$$

(7) $$s\left(k\right)={\left[s_{1}\left(k\right),s_{2}\left(k\right),\ldots ,s_{M}\left(k\right)\right]}^{T},$$

and

(8) $$n\left(k\right)={\left[n_{1}\left(k\right),n_{2}\left(k\right),\ldots ,n_{M}\left(k\right)\right]}^{T},$$

with dimensions M×M, M×1, and M×1, respectively.

The spatial correlation matrix of the array output is usually used for DOA estimation since it contains sufficient information about the received signals. From (5), the spatial correlation matrix, **R**, of the received noisy signals can be expressed as

(9) $$\begin{array}{cc} R & =E\left[y\left(k\right)y^{H}\left(k\right)\right]\\ \; & =\left[\begin{array}{cccc} E\left[y_{1}\left(k\right){y_{1}}^{H}\left(k\right)\right] & E\left[y_{1}\left(k\right){y_{2}}^{H}\left(k\right)\right] & \ldots & E\left[y_{1}\left(k\right){y_{M}}^{H}\left(k\right)\right]\\ E\left[y_{2}\left(k\right){y_{1}}^{H}\left(k\right)\right] & E\left[y_{2}\left(k\right){y_{2}}^{H}\left(k\right)\right] & \ldots & E\left[y_{2}\left(k\right){y_{M}}^{H}\left(k\right)\right]\\ \vdots & \vdots & \ddots & \vdots \\ E\left[y_{M}\left(k\right){y_{1}}^{H}\left(k\right)\right] & E\left[y_{M}\left(k\right){y_{2}}^{H}\left(k\right)\right] & \ldots & E\left[y_{M}\left(k\right){y_{M}}^{H}\left(k\right)\right] \end{array}\right]\\ & =\left[\begin{array}{cccc} R_{11} & R_{12} & \ldots & R_{1M}\\ R_{21} & R_{22} & \ldots & R_{2M}\\ \vdots & \vdots & \ddots & \vdots \\ R_{M1} & R_{M2} & \ldots & R_{MM} \end{array}\right], \end{array}$$

where $E\left[\cdot \right]$ is the statistical expectation operator, the superscript *H* denotes the complex conjugate transpose operation and $R_{ij}$ is the element in the *i*-th row and *j*-th column.

After applying the expectation operator, (9) can be rewritten as

(10) $$R=AR_{s}A^{H}+\sigma^{2}I,$$

where $\sigma^{2}$ represents the noise power (i.e., the variance) at the array elements, **I** is the identity matrix with dimensions *M* × *M*, and $R_{s}$ is the *K* × *K* signal correlation matrix that is written as

(11) $$R_{s}=E\left[s\left(k\right)s^{H}\left(k\right)\right].$$

Now the problem boils down to finding the angle of azimuth and elevation between the center of the receiving system and the source (i.e., ϕ, θ, respectively), having as input data the correlation matrix, **R**, of the receiving system. The correlation matrix can be unbiasedly estimated through the sample correlation matrix

(12) $$\bar{R}=\frac{1}{N-1}\sum_{k=1}^{N}y\left(k\right)y^{H}\left(k\right),$$

where *N* is the number of independent observations considered for calculating the matrix.

## 4. Proposed Method

**Figure 2:**
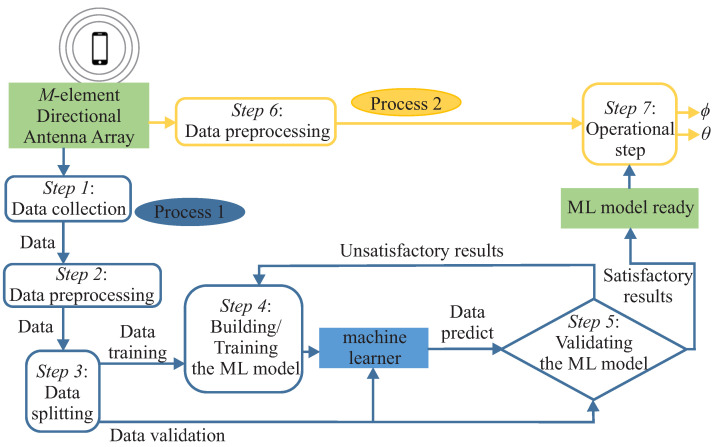
The System Model.

Figure 2 summarizes our proposal to find the azimuth and elevation angles of a source signal impinging on a receiver system. The proposed method will be divided into the ML model’s setup process (Process 1—blue colored boxes) and the up-and-running process (Process 2—yellow colored boxes). First, both processes are fed with the output of the antenna array in response to the source signal impinging on it. Next, the steps involved in these two processes are described.

*Step 1—Data collection*: This work uses supervised learning to find ϕ and θ angles. In supervised learning, the training data fed to the ML algorithm include pairs of fixed-dimension feature vectors and the desired output, called labels. In this step, the received signal obtained at the output of the antenna array **Y** and their respective labels are collected (azimuth and elevation angles that the transmitter forms with the center of the array). **Y** is the matrix formed by the *K* received signal vectors obtained at the output of the antenna array

(13) $$Y={\left[y(1),y(2),\ldots ,y(K)\right]}^{T},$$

where *K* is the number of collected vector samples.

In this work, we assume the size of the dataset is *L*. In that case, the fixed-dimensional feature vectors are composed of *L* spatial correlation matrices, $R_{l},\;\forall \;l\in \left\{1\ldots L\right\}$, which are converted into vectors, and the labels are their respective azimuth and elevation angles formed between the transmitter and the center of the receiving system (i.e., $\phi_{1}$, $\phi_{2}$, *…*, $\phi_{L}$ and $\theta_{1}$, $\theta_{2}$, *…*, $\theta_{L}$), where $\varphi_{1}$, $\varphi_{2}$, *…*, $\varphi_{L}$ are values between 0 and 2π radians and $\theta_{1}$, $\theta_{2}$, *…*, $\theta_{L}$ are values between 0 and π/2 radians.

*Step 2—Data preprocessing*: The preprocessing of the data is essential to achieve good performance with the ML algorithms. At this point, the *L* correlation matrices are calculated from the samples of the signals received by the receiving system, and their redundant information is removed. Since the correlation matrices $R_{l}$ are conjugate-symmetrical concerning their diagonal, the elements of their upper or lower triangular part provide enough information for 2D DOA estimation. This way, the other part of the matrix can be ignored. Therefore, next, we create feature vectors with the upper triangular part of the *L* correlation matrices, $R_{l}$, (which includes the main diagonal) with dimensions equal to $d_{R}=\sum_{i=1}^{M}i$.

In addition, the matrices $R_{l}$ contain complex values that cannot be interpreted by most ML models. Therefore, the matrices $R_{l}$ will be split into real and imaginary parts and arranged in a vector as follows

(14) rl=Re(R11,l)Re(R12,l)…Re(RMM,l)Im(R11,l)Im(R12,l)…Im(RMM,l),

where Re(Rij,l) and Im(Rij,l) are the real and imaginary parts of the element in the *i*-th row and *j*-th column of *l*-th correlation matrix, respectively. Thus, the dimensions of $r_{l}$ is $2d_{R}$. Notice that $r_{l}$ is the input data of the ML models.

*Step 3—Data splitting*: To validate the ML models, it is necessary to take part of the input data for training and another part, known as a validation set, usually smaller than the previous one, to evaluate the model when presented with unseen data, i.e., measure its generalization capacity. The validation set is used to evaluate the different metrics for the ML model by comparing the model’s predicted label with the actual value in the set. This way, it is possible to assess whether the model learned a general solution or not.

*Step 4—Building/Training the ML model*: In this step, the ML model is set up by choosing various strategies to improve the training results. First, we can select various ML regression models, for example, DT, Random Forest (RF), SVC, NN, etc. Specifically, in this work, we will work with the DT model. This model was chosen based on results obtained and published in [49]. DT models are one of the simplest yet most successful and powerful forms of ML models [50].

**Figure 3:**
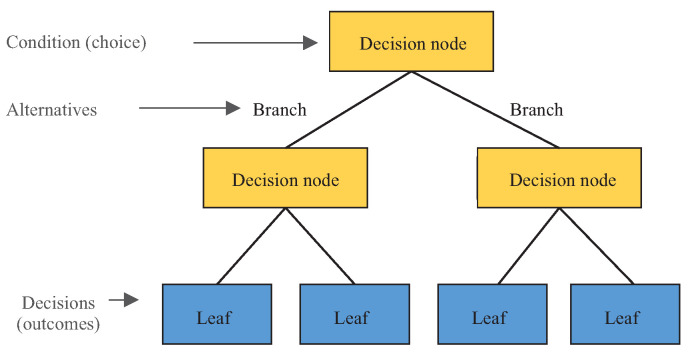
Structure of a Decision Tree.

**Figure 4:**
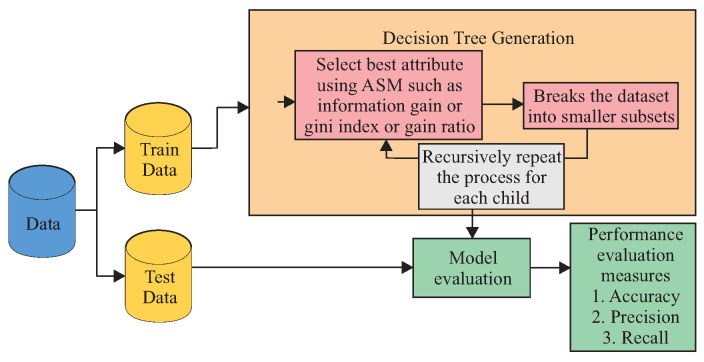
DT model: Process.

During the training phase, the DT algorithm builds (i.e., learns/trains) a tree-like model, as shown in Figure 3. The trained model is the one applied for making predictions, as shown in Figure 4. A DT is a tree-like structure where an internal node represents a feature (or attribute), the branch represents a decision rule, and each leaf node represents the outcome. The tree’s construction must start from a root node. The root node is the first node chosen by an Attribute Selection Measure (ASM) algorithm, such as information gain, gini index, and gain ratio. A typical decision tree evaluates the variable that best splits the data. Then, the model learns to partition based on the attribute value. Finally, it partitions the tree in a recursive way called recursive partitioning. Therefore, the construction of a DT can be summarized as described next.

1. Select the root node using ASM to split the records.
2. Make that attribute a decision node and break the dataset into smaller subsets.
3. Start building the tree by repeating this process recursively for each child until one of the following conditions matches:
  - All the tuples belong to the same attribute value.
  - There are no more remaining attributes.
  - There are no more instances.

Decision trees offer several advantages in data analysis. Firstly, they are simple to understand and interpret, and their structure can be visualized, providing a clear representation of the decision-making process. Additionally, decision trees require minimal data preprocessing, reducing the need for data normalization, dummy variables, and missing value handling. The computational cost of using decision trees is logarithmic in the number of data points used to train the tree, making predictions efficient even with large datasets. Furthermore, decision trees are versatile, capable of handling both numerical and categorical data, and they can also handle multi-output problems. Another advantage is that decision trees represent a white box model, where explanations for decisions are easily understood using boolean logic. This is in contrast to black box models like artificial neural networks, which can be more challenging to interpret. Additionally, decision trees enable the validation of the model using statistical tests, allowing for an assessment of the model’s reliability. Even when the assumptions of the true model are somewhat violated, decision trees tend to perform well in generating accurate predictions based on the given data.

This work uses the *sklearn.tree.DecisionTreeRegressor* [51] class from the *Scikit-Learn (sklearn)* library [52]. This class allows for Multi-Output Multi-Labels. This feature makes it possible for the proposed ML-based method to estimate azimuth and elevation angles simultaneously. Therefore, only one ML model is necessary for the DOA estimation. The *sklearn.tree.DecisionTreeRegressor* class takes as input the following parameters:

- *criterion*: The function to measure the quality of a split. This function can take the following values:
  – *squared_error*: for the mean squared error.
  – *friedman_mse*: uses mean squared error with Friedman’s improvement score for potential splits.
  – *absolute_error*: for the mean absolute error.
  – *poisson*: uses reduction in Poisson deviance to find splits.
- *splitter*: The strategy used to choose the split at each node. Supported strategies are *“best”* to choose the best split and *“random”* to choose the best random split.
- *max_depth*: This indicates how deep the tree can be.
- *min_samples_split*: The minimum number of samples required to split an internal node.
- *min_samples_leaf*: The minimum number of samples required at a leaf node.

The next step in the proposed method is hyperparameter optimization. The main goal of this optimization technique is to find a set of hyperparameters that optimizes the ML algorithm. Hyperparameter optimization reduces the human effort necessary for optimizing ML algorithms, improves their performance, and improves scientific studies’ reproducibility and fairness. There are different open-source software functions and libraries such as *sklearn.model_selection.GridSearchCV* [53], Talos [54], and Optuna [55] that facilitate the automation of hyperparameter optimization. In this work, we will employ Optuna for its simplicity and excellent results.

Optuna’s optimization algorithm is divided into sampling and pruning strategies, each playing a distinct role. The sampling strategy leverages Bayesian optimization [56], utilizing the historical data to determine the next set of hyperparameters or configurations. On the other hand, the pruning strategy enables faster optimization by identifying and discarding unpromising trials based on learning curves. Optuna offers numerous advantages that greatly influenced our decision to choose this tool. The key highlights of these advantages are outlined next.

- Lightweight, versatile, and platform-agnostic architecture that can be effortlessly integrated into various environments, allowing for easy adoption and usage.
- Handling a wide variety of hyperparameter optimization tasks, which offers flexibility and robustness to tackle various optimization scenarios effectively.
- Pythonic way of coding using familiar Python syntaxes, which simplifies the process of defining and exploring complex search spaces, enhancing user convenience and code readability.
- Efficient optimization algorithms, including state-of-the-art techniques for sampling hyperparameters and pruning unpromising trials, which lead to improved optimization performance and faster convergence toward optimal solutions.
- Easy parallelization, which allows the scaling of studies to tens or hundreds of workers with minimal or no code modifications, accelerating the optimization process, particularly when dealing with computationally intensive tasks.
- Quick visualization capabilities that enable swift inspection and analysis of optimization histories. It provides a range of plotting functions that allow for easy interpretation and understanding of the optimization process.

*Step 5—Validating the ML model*: After training the ML model, we validate it using part of the pre-processed data intended for this purpose (i.e., the *validation dataset*), that is, the part that was not used to train the ML model. For this, a prediction of the labels corresponding to the feature vectors of the validation set is made and compared with the actual labels. From here, we can obtain satisfactory or unsatisfactory results depending on whether the predicted labels are more or less similar to the actual ones. We analyze the results and adjust the ML model accordingly if the results are unsatisfactory.

There are different metrics to analyze how satisfactory the results of the ML model were. In this work, we will use the Root-Mean-Square Error (RMSE). The RMSE of an estimator measures the square root of the average of the squares of the errors, that is, the square root of the average of the squared difference between the estimated and the actual values. The RMSE for the proposed Multi-Output Multi-Label Proposal (MMP) is calculated as follows

(15) $$RMSE\left(\phi ,\hat{\phi },\hat{\theta },\theta \right)=\sqrt{\frac{1}{2L}\sum_{i=0}^{L-1}{\left({\hat{\phi }}_{i}-\phi_{i}\right)}^{2}+{\left({\hat{\theta }}_{i}-\theta_{i}\right)}^{2}},$$

where *L* is the number of examples, ϕ, θ are the actual azimuth and elevation angles, and $\hat{\phi }$, $\hat{\theta }$ are the azimuth and elevation angles predicted by the ML model, respectively.

*Step 6—Data preprocessing*: The same pre-processing as the one described in Step 2 is carried out for new data samples collected during the operational use of the proposed DOA estimation method.

*Step 7—Operational step*: Once satisfactory results are obtained, the trained and optimized ML model can be used to predict the azimuth and elevation angles of new data samples.

## 5. Experimental Results

In this section, we describe how the dataset was generated and present several experimental results used for assessing the proposed DOA estimation method. In the first part, we discuss how the dataset was created. Next, we analyze the robustness of the method. Finally, in the third part, we compare it with a well-known DOA estimation algorithm.

All receiving models are simulated in Matlab, and the collected information is used to create the dataset. The Python language and the Scikit-Learn library are used to implement the DT-based DOA estimation method. All experiments are performed on an Acer laptop with a Core i7 processor, 16 GB of RAM memory, and an 8 GB Intel(R) Iris(R) Xe graphics video card.

### 5.1. Data Generation

Based on the system model described in Section 3 and using the Matlab simulation tool, we generated the dataset that was used for training and validating the proposed DT-based DOA estimation method. Matlab was also used to generate the radiation patterns of the half-wavelength strip dipole antennas.

For each pair of azimuth and elevation angles, i.e., ($\phi_{i}$, $\theta_{i}$), the corresponding *l*-th correlation vector, $r_{l}$, was obtained. The azimuth and elevation angles are in the range [0°, 360°) and [0°, 90°), respectively, with an angular resolution of 1°. For each angle pair, 20 correlation vectors, $r_{l}$, were generated. Each correlation vector corresponds to a realization of the AWGN channel. This way, the total number of correlation vectors, $r_{l}$, generated is equal to 648,000, i.e., 20 noise realizations × 360 azimuth angles × 90 elevation angles. It is assumed that s(k)=1, ∀*k*, and $n_{m}\left(k\right)$ is circularly symmetric, independent, and identically distributed complex AWGN with zero mean and variance in the range $10^{-4}$ to 10. Moreover, we are going to compare the proposed method for a different number of receiving antennas, M= 4, 8, and 12.

### 5.2. Analysis of the Robustness of the ML Model

In this section, we assess the robustness of the ML model against variations in the received signal. Let us suppose all data in the collected database have the same noise variance. In that case, there is a significant probability that when the received signal has a different noise variance, the model will predict the wrong angles because it was trained with a dataset generated with signals having a different noise variance. For example, if the DT model is trained with correlation vectors of signals with an SNR of −10 dB, the model could incorrectly predict the azimuth and elevation angles when the received signal has an SNR of 20 dB. Next, we describe two experiments that will be used to analyze whether the DT model can correctly predict the angles using signals with different SNRs during the training and prediction stages.

- *Experiment 1*: For a given number of receiving antennas, *M*, one single DT model is trained with a dataset comprising correlation vectors of signals of all SNR values in the set −10, 0, 10, 20, 30, and 40 dB. Subsequently, also for a specific number of receiving antennas, the model is validated with datasets composed of correlation vectors of each individual SNR value. This training and validation process is repeated for each different number of receiving antennas considered (M= 4, 8, and 12).
- *Experiment 2*: For a given number of receiving antennas, *M*, different DT models are trained with a dataset containing correlation vectors of one specific SNR value in the set −10, 0, 10, 20, 30, and 40 dB. Subsequently, also for a specific number of receiving antennas, the models are validated with datasets composed of correlation vectors of each individual SNR value. This training and validation process is repeated for each different number of receiving antennas considered (M= 4, 8, and 12).

#### 5.2.1. Experiment 1—Results

To perform this experiment, 19 examples of each combination of angles and SNR values were used for training purposes. The azimuth angles are between 0° and 359°, and the elevation angles range from 0° to 89° with a resolution of 1°. One DT model was trained with a dataset composed of correlation vectors with SNR values of −10, 0, 10, 20, 30, and 40 dB. In other words, 3,693,600 (i.e., 19 × 360 × 90 × 6) samples (i.e., correlation vectors) were used for training the DT model. On the other hand, one example of each combination of angles and SNR value was used to validate the model for each SNR value, generating six datasets with 32,400 (i.e., 1 × 360 × 90) samples each. Optuna was employed to optimize the hyperparameters of the DT model. This process (i.e., training, optimization, and validation) was repeated for each number of receiving antennas in the set M= 4, 8, and 12. The obtained hyperparameter optimization results for all antenna numbers are presented in Table 2.

**Figure 5:**
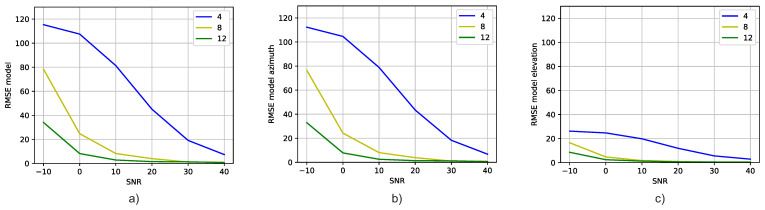
Comparison of the RMSE for different numbers of receiving. (**a**) Azimuth and elevation angles’ RMSE versus SNR for different antenna numbers, (**b**) Azimuth angle’s RMSE versus SNR for different antenna numbers, and (**c**) Elevation angle’s RMSE versus SNR for different antenna numbers.

Figure 5 shows the RMSE versus SNR values attained by the DT models trained with correlation vectors containing all SNR values and for different numbers of receiving antennas. The models were validated with six datasets, each one comprising correlation vectors with a unique SNR value. Figure 5a shows the RMSE of the DT models when simultaneously predicting the azimuth and elevation angles. Figure 5b,c show the RMSE of the DT models when predicting the azimuth and elevation angles, respectively.

Figure 5 as a whole shows that the DT model’s estimation performance improves as the number of antennas increases. This is expected, since the available correlation information increases. Moreover, the figure also shows that training the DT model with a dataset comprised of correlation vectors with different SNRs demonstrates good performance.

**Figure 6:**
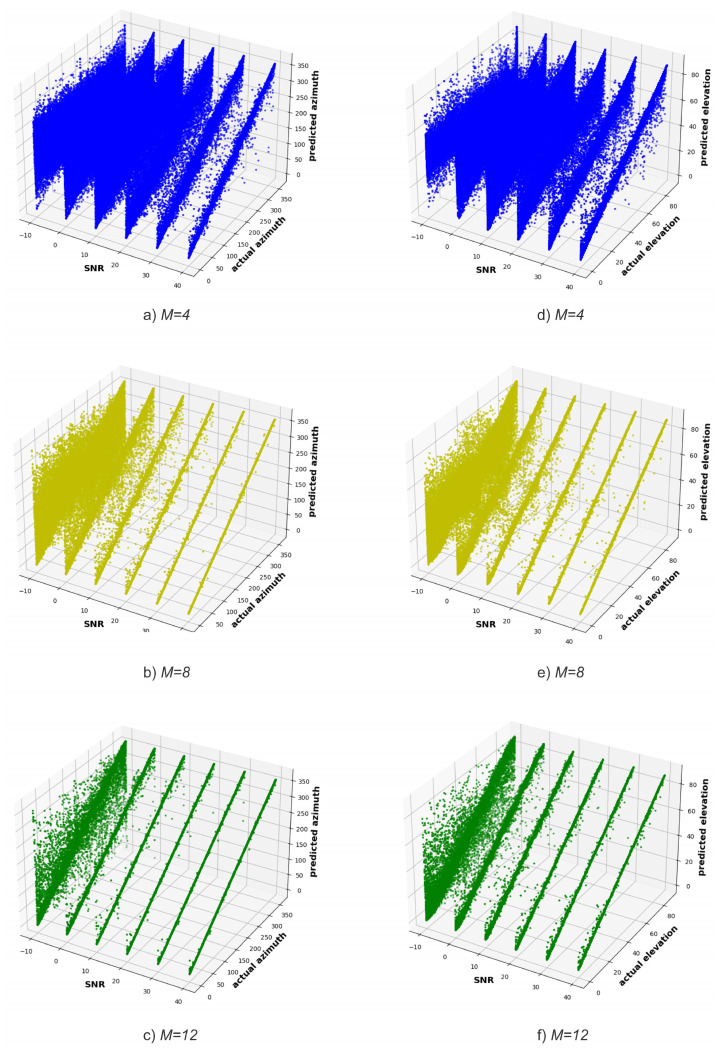
SNR versus actual and predicted azimuth angle for (**a**) *M* = 4, (**b**) *M* = 8, (**c**) *M* = 12, and elevation angle for (**d**) *M* = 4, (**e**) *M* = 8, (**f**) *M* = 12 (Experiment 1).

Figure 6 shows the SNR versus the actual and predicted angles for ϕ (Figure 6a–c) and θ (Figure 6d–f), respectively. As the number of antennas of the receiving system increases, the diagonal becomes narrower (i.e., less noisy), both in the prediction of ϕ and θ angles, which means a greater correspondence between the actual and predicted angles. Furthermore, for the same number of receiving system antennas, the sharpness of the diagonal improves as the SNR increases.

In addition, when looking at Figure 6 and comparing the predictions of ϕ and θ angles, one might be made to believe that the model better predicts the ϕ angle, which is contradicted by the results in Figure 5b,c. This happens because θ angles are in a shorter interval, [0°, 90°), than ϕ angles, [0°, 360°). As a matter of fact, in order to plot the results shown in Figure 6, the DT model performs the same number of predictions for θ and ϕ angles. This means that the DT model has more information to learn from in order to estimate the elevation angle compared to the azimuth angle, making it easier for the model to estimate elevation angles than the azimuth ones. Therefore, when plotting the same number of points (i.e., predictions) in a smaller space, the points seem more dispersed, and consequently, the diagonal seems less narrow (i.e., is less defined) when compared with the diagonal of the azimuth angles.

#### 5.2.2. Experiment 2—Results

To perform this experiment, 19 examples of each combination of angles and one specific SNR value were used to train one model. In total, 615,600 (19 × 360 × 90) samples of one given SNR value were used to train the DT models. An example of each combination of angles and specific SNR was used to validate the models.

As in Experiment 1, we first use Optuna to optimize the hyperparameters of the models. Since we want to train DT models for different numbers of antennas and specific SNR values, we are going to have a total of 18 models (i.e., models for 4, 8, and 12 antennas that are trained with datasets composed of six different SNR values, −10, 0, 10, 20, 30, and 40 dB). The obtained hyperparameter optimization results for all SNR values and antenna numbers are presented in Table 3.

**Figure 7:**
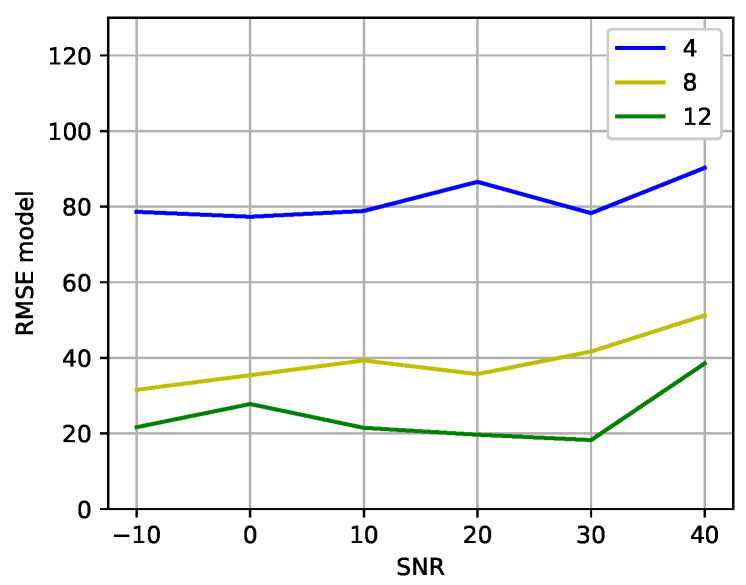
Average RMSE of the DT models versus the SNR of the signals used to train the models.

Figure 7 shows the results of training the DT models with correlation vectors of the received signals. Each model was trained with a dataset composed of correlation vectors of signals with one specific SNR and then validated with correlation vectors of signals with different SNRs. The x-axis shows the SNR of the signals with which the DT model was trained, and the y-axis shows the average RMSE of validating the DT models with signals with SNRs of −10, 0, 10, 20, 30, and 40 dB. When analyzing the results shown in Figure 7, it is observed that, globally, particularly for M>4, the DT models exhibit improved capabilities in predicting azimuth and elevation angles of signals with varying SNRs when trained with signals possessing an SNR of approximately 20 dB. Therefore, next, we use models trained with signals having an SNR of approximately 20 dB.

**Figure 8:**
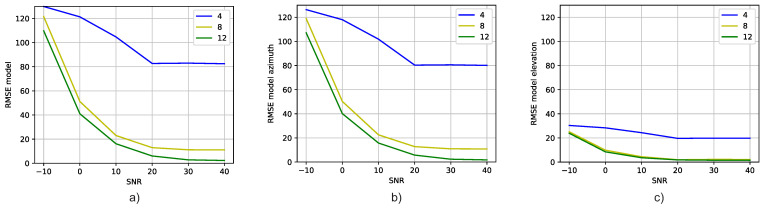
Comparison of the RMSE of DT models trained with signals having an SNR of 20 dB versus the SNR of the signals used to validate them: (**a**) Azimuth and elevation angles’ RMSE versus SNR for different antenna numbers, (**b**) Azimuth angle’s RMSE versus SNR for different antenna numbers, and (**c**) Elevation angle’s RMSE versus SNR for different antenna numbers.

**Figure 9:**
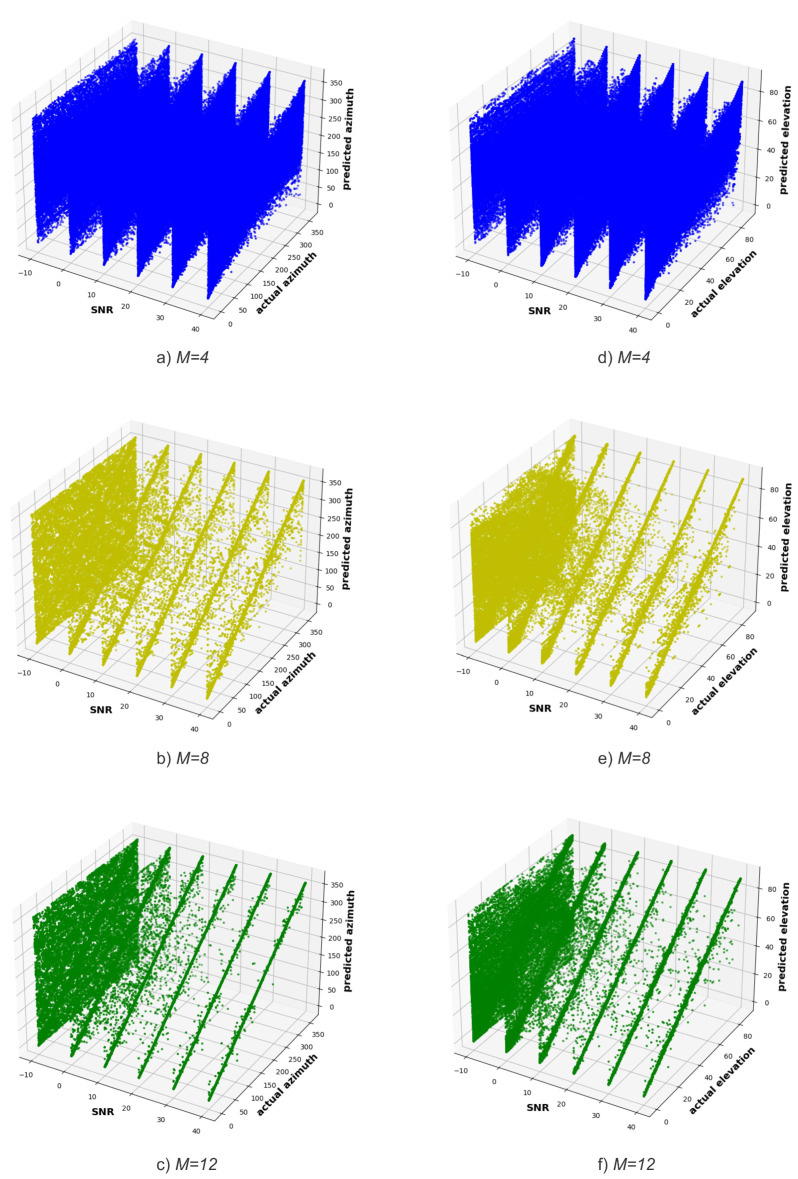
SNR versus actual and predicted azimuth angle for (**a**) *M* = 4, (**b**) *M* = 8, (**c**) *M* = 12, and elevation angle for (**d**) *M* = 4, (**e**) *M* = 8, (**f**) *M* = 12 (Experiment 2).

Figure 8 displays the validation results of DT models trained with correlation vectors presenting an SNR of 20 dB. The validation is performed using correlation vectors presenting SNR values ranging from −10 dB to 40 dB (x-axis). The graph illustrates that training the DT model with correlation vectors of signals at an SNR of 20 dB and validating it with SNR signals from −10 dB to 40 dB yields satisfactory performance. Moreover, Figure 9 demonstrates consistent predictions of azimuth and elevation angles when training the model with signals at an SNR of 20 dB. Once more, we see that the diagonal becomes narrower as the number of antennas and SNR increase.

#### 5.2.3. Comparison between Experiments 1 and 2

Let us delve into the comparison of the results obtained from experiments 1 and 2 (i.e., Figure 5 and Figure 8, respectively). Upon careful analysis, it becomes apparent that the results of experiment 1 outshine those achieved in experiment 2. Notably, experiment 1 showcases a smaller RMSE for both low SNR values and a limited number of antennas, as small as four. This compellingly signifies that training a DT model with a diverse dataset containing correlation vectors encompassing various SNR values proves to be a superior approach compared to training the model with signals having a specific SNR value.

These results highlight the efficacy of employing DT models in accurately estimating azimuth and elevation angles across a wide range of SNR values. While experiment 1 outperformed experiment 2 in terms of RMSE, both experiments demonstrate the overall efficiency and robustness of proposals utilizing DT models in handling a broad range of SNR values. This reaffirms the potential of DT-based methodologies for accurate angle estimation, providing valuable insights into their application in real-world scenarios.

### 5.3. Comparison with MUSIC

**Figure 10:**
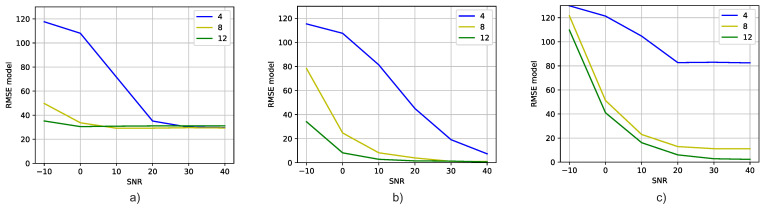
Comparison of the RMSE vs. SNR for different antenna numbers: (**a**) MUSIC, (**b**) Experiment 1, (**c**) Experiment 2.

**Figure 11:**
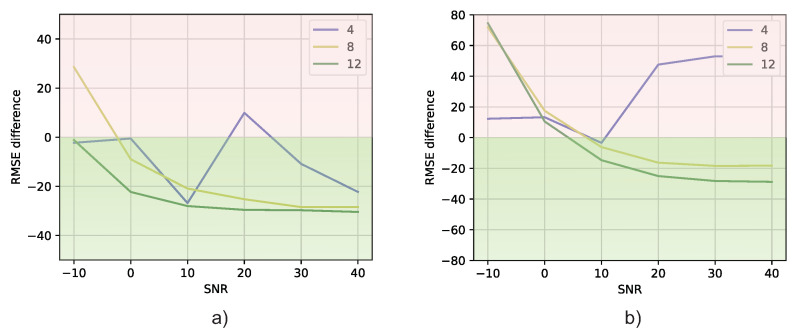
Difference between the RMSE of the DT and MUSIC models for each experiment and SNR value: (**a**) Experiment 1, (**b**) Experiment 2.

Finally, let us compare the results of the well-known MUSIC DOA estimation algorithm with our proposed method [19]. In this work, we use the Matlab implementation of the algorithm [57]. In Figure 10a, we observe the MUSIC results for the resolution of the same azimuth and elevation angles as resolved by the DT model. Comparing the results of MUSIC with those of Experiments 1 and 2 (Figure 10b,c, respectively), it becomes apparent that, on the whole, the DT model achieves superior performance. Furthermore, Figure 11 presents the difference between the RMSE of the DT and MUSIC models for each SNR value. This difference is calculated as follows

(16) $$RMSE_{difference}=RMSE_{DT}\left(SNR\right)-RMSE_{MUSIC}\left(SNR\right).$$

Thus, if the RMSE difference is less than zero (green color in the figure), it means that the DT model is closer to the expected value of DOA than MUSIC. On the other hand, if the RMSE difference is positive (red color in the figure), it means that MUSIC obtains a better result.

Comparing Figure 10 and Figure 11, it can be seen that, in general, the DT models are closer to the expected azimuth and elevation angles than MUSIC. It is mainly true for Experiment 1 (Figure 11a). For Experiment 2, MUSIC obtains better results across almost all SNR values when the number of receiver antennas is four. In addition to this analysis, a statistical analysis for the difference between the results of the MUSIC and DT models using the Wilcoxon signed-rank test was carried out [58]. With this analysis, we obtained $p_{value}=0.00438$. Therefore, the difference between the DOA estimation methods is significant, since the $p_{value}$ is less than the significance level of 0.05. Therefore, it is concluded that there is enough evidence to state that the methods are really different, with the DT being the best one in terms of the RMSE.

The comparison between the DT model and the MUSIC algorithm underscores the advantages of our approach in achieving accurate DOA estimation. The DT model outperforms MUSIC in terms of RMSE, especially for signals with higher SNR values. Additionally, the DT model showcases the ability to leverage an increasing number of antennas to enhance accuracy, which is not observed in the case of MUSIC.

**Figure 12:**
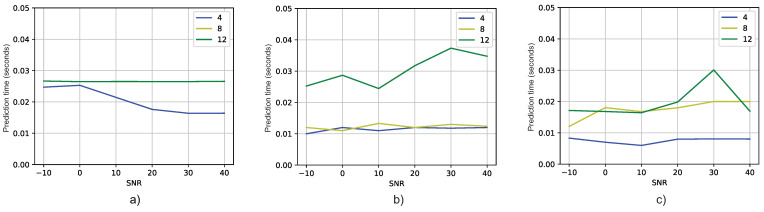
Prediction time vs. SNR for different antenna numbers: (**a**) MUSIC, (**b**) Experiment 1, (**c**) Experiment 2.

Next, let us examine Figure 12a, which displays the average time taken by the MUSIC algorithm to estimate the azimuth and elevation angles. Additionally, Figure 12b,c, illustrate the average time consumed by the DT models in Experiments 1 and 2, respectively. Upon comparing these figures, it becomes apparent that, in general, the average prediction times of DT models are shorter than the average prediction time of the MUSIC algorithm.

As in the case of RMSE, we wanted to analyze how different the DT models were from the MUSIC models in terms of prediction time, i.e., the time it takes for the model to output an estimate. The prediction difference is calculated as the DT’s prediction time minus that of MUSIC, which is represented by (17).

(17) $$Time_{difference}=Time_{DT}\left(SNR\right)-Time_{MUSIC}\left(SNR\right).$$

**Figure 13:**
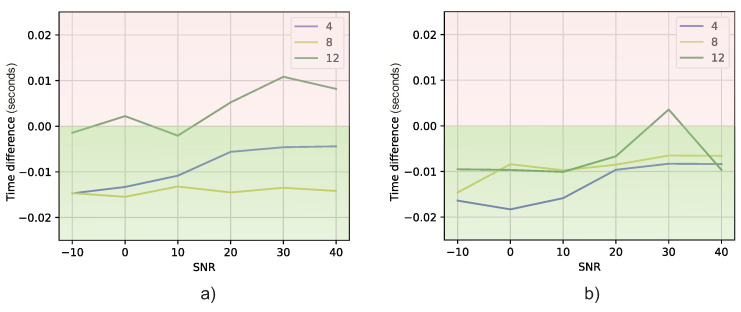
Time difference versus SNR: (**a**) Experiment 1 DT models in respect to MUSIC method, (**b**) Experiment 2 DT models in respect to MUSIC method.

The prediction difference between both models is presented in Figure 13. As in Figure 11, if the results are below zero, it means that the DT model is faster than MUSIC. In Figure 13, it is shown that, indeed, for most of the cases, the DT models predict the angles faster than MUSIC. In addition, according to the Wilcoxon signed-rank test theorem, this difference is significant, since $p_{value}=0.01078$ ($p_{value}<0.05$). Therefore, as before, it is concluded that there is enough evidence to state that the methods are really different, with the DT being the faster one in terms of the prediction time.

This finding not only reinforces the superior accuracy demonstrated by DT models, as discussed earlier in terms of RMSE results, but also highlights their efficiency in terms of prediction time. The reduced prediction times indicate that DT models offer a faster computational approach to achieving accurate DOA estimation when compared to the conventional MUSIC method.

By excelling in both RMSE and computational efficiency, our model exhibits a clear advantage over the MUSIC algorithm. These results validate the effectiveness of the DT models, providing a solid foundation for their practical implementation in real-world scenarios where timely and precise DOA estimation is of utmost importance.

## 6. Limitations of the Proposed Method

In this section, we describe some limitations the proposed method presents.

- *Generalizability*: The study primarily investigates a specific scenario involving line-of-sight communication between a single transmitter and the receiving system. Therefore, the diversity of the training dataset might not cover all possible real-world scenarios, potentially affecting the method’s performance in certain situations. The method’s accuracy and generalization capability may vary when applied to more complex scenarios. As such, the direct applicability of the findings to other contexts may be restricted. The generalizability of the results is more likely to be applicable to similar scenarios and related applications. In future research, it is important to consider additional scenarios, such as those involving fading channels and simultaneously transmitting devices. By incorporating these varied scenarios, a more comprehensive understanding of the subject matter can be achieved, leading to broader applicability and enriched insights.
- *Exploration of ML models*: The study exclusively utilizes DT models owing to their simplicity, low complexity, and superior performance when compared to more intricate models such as neural networks. However, this approach imposes a limitation by precluding the exploration of potentially superior models. Future research endeavors should encompass a broader spectrum of ML models, such as neural networks, support vector machines, or ensemble methods, to facilitate comprehensive comparisons and gain insights into their respective strengths and weaknesses when tackling the specific problem at hand. By incorporating these diverse models, the analysis can be enriched, leading to a deeper understanding and improved overall assessment.
- *Antenna Array Configuration*: The performance of the DT-based method could be influenced by the specific antenna array configuration used in the study. Different antenna array configurations may yield varying results, and the effectiveness of the model may depend on the physical setup of the array.
- *Real-time constraints*: The present research does not take real-time processing requirements and analysis into account, which is a crucial aspect in certain applications. The lack of real-time processing assessment in the study might limit its applicability to time-sensitive scenarios. Future investigations should consider incorporating real-time considerations to render the proposed methodologies suitable for real-world applications.
- *Simulation-based results*: The results are based on simulation studies, which may not perfectly reflect real-world conditions. The model’s performance in an actual implementation could differ from the simulation results due to factors such as noise, interference, and other real-world complexities.

It is important to acknowledge these limitations to provide a clear understanding of the study’s scope and potential implications. Addressing these limitations in future research will contribute to a more comprehensive and nuanced understanding of the problem under investigation.

## 7. Conclusions

In conclusion, DOA estimation has been a widely researched topic in signal processing, with applications spanning various fields such as beamforming, localization in 5G and 6G networks, drone localization, radar, sonar, seismology, astronomy, and military surveillance, among others.

Therefore, by recognizing the significance of DOA estimation, this study has proposed and analyzed a novel method based on DTs for accurately determining the azimuth and elevation angles of signals impinging on a receiving antenna array system. The primary objective of this proposed method is to improve the accuracy, efficiency, and applicability of DOA estimation.

The DT models employed in this method are capable of estimating the azimuth and elevation angles between a transmitter and a system receiver. They utilize information provided by the correlation matrix derived from the signals received by an antenna system. To enhance the training and prediction processes of the DT models, their hyperparameters have been optimized using the Optuna framework.

Two experiments were conducted to demonstrate the effectiveness of the proposed solution in predicting the azimuth and elevation angles under different conditions from those used during the training of the ML model. The results from both experiments indicate that the DT models employed in the proposed method can effectively learn to predict azimuth and elevation angles with signals characterized by varying SNRs. When compared to the MUSIC DOA estimation algorithm, the proposed solution exhibited significantly better results in minimizing both the RMSE and the average prediction time. In a more quantitative way, the results of the conducted experiments show a significant average reduction of more than 90% in the prediction error and 50% in the prediction time achieved by the proposed DT-based DOA estimation method when compared to the MUSIC algorithm.

In future research, it would be valuable to explore and evaluate the performance of alternative types of receiving systems and ML models, including neural networks. Furthermore, the proposal presented in this study, as well as future ones, should be validated through real-world experiments to complement the findings obtained from simulations. Such real experiments would provide further validation and ensure the practical viability of the proposed solutions.

## Figures and Tables

**Table 1:** Taxonomy of the proposed ML to DOA estimation.

Ref	Azimuth	Elevation	Single-Source	Multi-Source	Simulation	Experiment	Comparative with Other Works
[28]	x			x	x	x	x
[29]	x		x		x	x	x
[30]		x		x	x		
[31]	x			x	x		x
[32]		x		x	x		x
[33]	x		x		x		x
[34]	x		x			x	
[35]	x	x	x		x		x
[36]			x		x	x	x
[37]		x	x	x	x		x
[38]		x	x	x		x	x
[39]	x	x	x	x	x		x
[40]		x		x	x		
[41]		x		x	x		x
[42]	x		x		x		x

**Table 2:** Summary of the best model hyperparameters for Experiment 1.

Parameters	*max_depth*	*min_samples_split*	*min_samples_leaf*	*splitter*	*criterion*	*max_features*
Selection range	100–1100, step: 100	2–40	1–40	*“best”, “random”*	*“friedman_mse”, “poisson”*	*“auto”, “log2”, “sqrt”*
M=4	500	27	19	*“best”*	*“friedman_mse”*	*“auto”*
M=8	700	16	21	*“best”*	*“friedman_mse”*	*“log2”*
M=12	1000	5	34	*“random”*	*“friedman_mse”*	*“auto”*

**Table 3:** Summary of the best model hyperparameters for Experiment 2.

	Parameters	*max_depth*	*min_samples_split*	*min_samples_leaf*	*splitter*	*criterion*	*max_features*
	Selection Range	100–1100, Step: 100	2–40	1–40	*“best”, “random”*	*“friedman_mse”, “poisson”*	*“auto”, “log2”, “sqrt”*
*SNR = −10 dB*	M=4	400	16	34	*“best”*	*“friedman_mse”*	*“log2”*
	M=8	500	27	6	*“random”*	*“friedman_mse”*	*“sqrt”*
	M=12	800	35	23	*“random”*	*“friedman_mse”*	*“sqrt”*
*SNR = 0 dB*	M=4	800	19	17	*“random”*	*“friedman_mse”*	*“auto”*
	M=8	100	3	12	*“random”*	*“friedman_mse”*	*“sqrt”*
	M=12	900	28	24	*“random”*	*“friedman_mse”*	*“log2”*
*SNR = 10 dB*	M=4	600	7	13	*“best”*	*“friedman_mse”*	*“log2”*
	M=8	900	22	22	*“random”*	*“friedman_mse”*	*“log2”*
	M=12	200	11	12	*“best”*	*“friedman_mse”*	*“log2”*
*SNR = 20 dB*	M=4	500	12	10	*“random”*	*“friedman_mse”*	*“sqrt”*
	M=8	200	39	9	*“random”*	*“friedman_mse”*	*“log2”*
	M=12	400	22	8	*“best”*	*“friedman_mse”*	*“logs2”*
*SNR = 30 dB*	M=4	900	4	16	*“best”*	*“friedman_mse”*	*“log2”*
	M=8	1000	16	37	*“random”*	*“friedman_mse”*	*“log2”*
	M=12	400	35	4	*“best”*	*“friedman_mse”*	*“sqrt”*
*SNR = 40 dB*	M=4	400	14	23	*“best”*	*“poisson”*	*“sqrt”*
	M=8	800	37	38	*“random”*	*“poisson”*	*“sqrt”*
	M=12	100	34	25	*“random”*	*“poisson”*	*“sqrt”*

## Data Availability

Not applicable.

## Abbreviations

The following abbreviations are used in this manuscript:

ML	Machine Learning
DT	Decision Tree
DOA	Direction of Arrival
BF	Beamforming
IoT	Internet of Things
UAV	Unmanned Aerial Vehicle
MLE	Maximum Likelihood Estimator
SBT	Subspace-Based Techniques
RSS	Received Signal Strength
CRB	Cramer-Rao Bound
MUSIC	Multiple Signal Classification
ESPRIT	Estimation of Signal Parameters Via Rotational Invariance Techniques
SSR	Sparse Signal Reconstruction
NN	Neural Network
CVNN	Complex Valued Neural Network
RVNN	Real-Valued Neural Network
MPNN	Multilayer Perceptron Neural Network
GCC	Generalized Cross-Correlation Vectors
DNN	Deep Neural Network
ULA	Uniform Linear Array
MIMO	Multiple Input Multiple Output
CNN	Convolutional Neural Network
MSE	Mean Squared Error
SVD	Singular Value Decomposition
CFCN	Circularly Fully Convolutional Networks
SVR	Support Vector Regression
FBLP	Forward–Backward Linear Prediction
SVM	Support Vector Machine
AWGN	Additive White Gaussian Noise
HPO	Automated Hyperparameter Optimization
RMSE	Root-Mean-Square Error
MMP	Multi-Output Multi-Label Proposal
UCA	Uniform Circular Array
SNR	Signal-to-Noise Ratio

## Symbols

The following symbols are used in this manuscript:

$\ell_{p}$-norms	Space function.
$\ell_{1}$-norm	The sum of the magnitudes of the vectors in space.
*M*	A number of antennas of the receiving system.
*r*	UCA radius.
λ	Incident signal’s wavelength.
ϕ	Azimuth angle.
θ	Elevation angle.
*m*	Antenna number index.
$y_{m}\left(k\right)$	Received signal at the *m*-th antenna element.
$\gamma_{m}$	The angular position of the *m*-th antenna element.
$a_{m}\left(\phi ,\theta ,\gamma_{m}\right)$	Attenuation factor.
s(k)	Signal transmitted by the source.
$n_{m}\left(k\right)$	Complex Additive White Gaussian Noise at the *m*-th antenna element.
*k*	Sample index.
*K*	Number of collected vector samples at the output of the antenna array.
$\sigma^{2}$	The variance of Additive White Gaussian Noise.
y(k)	Received signal vector obtained at the output of the antenna array.
**A**	Matrix of the attenuation factor.
**s**(k)	Vector of the signal transmitted by the source.
**n**(k)	Vector of Complex Additive White Gaussian Noise at the output of the antenna array.
${\left[\cdot \right]}^{T}$	Transpose of a matrix
**R**	Spatial covariance matrix.
$E\left[\cdot \right]$	Statistical expectation operator.
*H*	Complex conjugate transpose operation.
**I**	Identity matrix with dimensions *M* × *M*.
*N*	Number of independent observations considered for calculating the **R** matrix.
Y	Matrix formed by the *K* received signal vectors
	obtained at the output of the antenna array.
*L*	Size of the dataset used to train ML models.
$R_{l}$	Correlation matrix.
$r_{l}$	Vector formed by the real and imaginary parts of each element $R_{ij,l}$ of the $R_{l}$ matrix.
Re(Rij,l)	Real part of the element in the *i*-th row and *j*-th column of $R_{l}$ matrix.
Im(Rij,l)	Imaginary part of the element in the *i*-th row and *j*-th column of $R_{l}$ matrix.
$\hat{\phi }$	Azimuth angle predicted by the ML model.
$\hat{\theta }$	Elevation angle predicted by the ML model.
